# *Euphorbia formosana* Root Extract Induces Apoptosis by Caspase-Dependent Cell Death via Fas and Mitochondrial Pathway in THP-1 Human Leukemic Cells

**DOI:** 10.3390/molecules18021949

**Published:** 2013-02-01

**Authors:** Yi-Jen Hsieh, Chih-Jui Chang, Chin-Feng Wan, Chin-Piao Chen, Yi-Han Chiu, Yann-Lii Leu, Kou-Cheng Peng

**Affiliations:** 1 Department of Laboratory Medicine and Biotechnology, School of Medicine, Tzu Chi University, Hualien 97004, Taiwan; 2 Department of Chemistry, National Dong-Hwa University, Hualien 97401, Taiwan; 3 Department of Molecular Biology and Human Genetics, Tzu Chi University, Hualien 97004, Taiwan; 4 School of Applied Chemistry, Chung Shan Medical University, Taichung 40201, Taiwan; 5 Institute of NanoEngineering and MicroSystems, National Tsing Hua University, Hsinchu 30013, Taiwan; 6 Department of Nursing, St. Mary’s Medicine, Nursing and Management College, Yi-Lan 26644, Taiwan; 7 Graduate Institute of Natural Products, College of Medicine, Chang Gung University, Taoyuan 333, Taiwan; 8 Chinese Herbal Medicine Research Team, Healthy Aging Research Center, Chang Gung University, Taoyuan 333, Taiwan; 9 Institute of Biotechnology, National Dong-Hwa University, Hualien 97401, Taiwan

**Keywords:** *Euphorbiaformosana*, apoptosis, mitochondrial pathway, Fas pathway

## Abstract

Acute myeloid leukemia (AML), a very rare type of cancer, generally affects patients over 50 years old. While clinical drugs to treat advanced stages of AML exist, the disease becomes increasingly resistant to therapies. *Euphorbia* *formosana* Hayata (EF) is a native Taiwanese medicinal plant used to treat rheumatism, liver cirrhosis, herpes zoster, scabies, and photoaging, along with tumor suppression. However, the mechanisms by which it suppresses tumors have not been explored. Here, we provide molecular evidence that a hot-water extract of *Euphorbia* *formosana* (EFW) selectively inhibited the growth of human leukemic cancer cells more than other solid human cancer cell lines. Most importantly, the plant extract had limited toxicity toward healthy peripheral blood mononuclear cells (PBMCs). After THP-1 leukemic cells were treated with 50–100 µg/mL EFW for one day, the S phase DNA content of the cells increased, while treatment with 200–400 µg/mL caused the cells to accumulate in the G0/G1 phase. Notably, EFW did not affect A-549 lung cancer cells. The effectiveness of EFW against THP-1 cells may be through caspase-dependent apoptosis in leukemic cells, which is mediated through the Fas and mitochondrial pathways. The potent antileukemic activity of EFW *in vitro* warrants further investigation of this plant to treat leukemias and other malignancies.

## 1. Introduction

Acute myeloid leukemia (AML) is characterized by the rapid growth of abnormal white blood cells, which accumulate in the bone marrow and interfere with the production of normal blood cells. About 80% of adult cases of acute leukemia are caused by AML, which is the most common type of acute leukemia in adults. Untreated AML is fatal within weeks or months, since the disease leads to marrow failure and death [1]. In the United States, the annual incidence of AML is approximately 2.4 cases per 100,000 adults, though the incidence increases progressively with age and peaks at 12.6 cases per 100,000 adults of age 65 or older. Leukemic cells replace normal bone marrow in AML, leading to dropping counts of red blood cells, platelets, and normal white blood cells. Symptoms of AML include fatigue, shortness of breath, easy bruising, easy bleeding, and increased risk of infection. Though several risk factors and chromosomal abnormalities have been identified, the specific cause is not clear. The primary treatment for AML is chemotherapy such as idarubicin, which induces cell death through apoptosis that results from intrinsic mitochondrial or extrinsic death-receptor pathways. Patients treated with chemotherapy achieve complete but transient remissions, and then develop resistance when their disease relapses. Clearly, AML patients would benefit from therapies that are free from relapses. 

Among natural sources, many effective anticancer agents have come from natural products from plants [2,3,4,5,6,7,8,9,10]. Botanical medicines are generally plentiful, low cost, and relatively non-toxic in clinical practice. In many cases, plant extracts are thought to be therapeutically superior to their single isolated constituents [11]. So, their medicinal properties are under extensive investigation, as their use has become a major part of complementary and alternative medicines (CAMs). 

*Euphorbia formosana* (EF) is a Taiwanese native medicinal plant that is used to treat rheumatism, liver cirrhosis, herpes zoster, scabies, and photoaging [12], though not leukemia. The ability of EF to mediate proapoptotic activity intrigued us to explore its possible applications as CAM for AML. Additionally, understanding the molecular targets and mechanism of action of EF will enable combination therapies to be rationally designed to more efficiently eradicate leukemic cells. This research aims to evaluate EF extract that is produced by artificial cuttage for efficacy on various leukemic cell lines and to understand its working mechanisms. We have found that EF specifically induces apoptosis in various leukemic cell lines. 

## 2. Results

### 2.1. EFW Specifically Inhibits the Growth of Leukemic Cancer Cells

**Figure 1:**
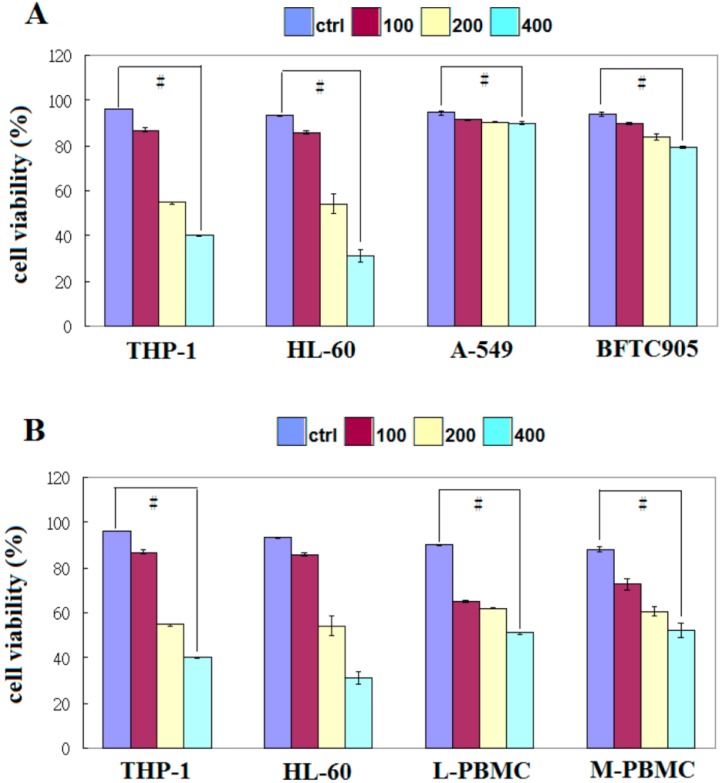
(**A**) The effect of 24 h of treatment with EFW at 100 μg/mL (red), 200 μg/mL (yellow), and 400 μg/mL (green) or with distilled water (vehicle control; blue) on the growth of THP-1, HL-60, A-549, BFTC905, and PBMCs; (**B**) The effect of EFW on the cell proliferation of THP-1, HL-60, L-PBMC, and M-PBMC cells was analyzed by flow cytometry. The data represent the mean ± S.D. of three independent experiments, and the symbol # indicates significance of *p* < 0.001 compared with the control experiments.

We analyzed the effects of EFW on cell proliferation by treating several carcinoma cell lines with three different concentrations of EFW. Both leukemic cell lines, THP-1 and HL-60, had dose-dependent growth inhibition of 40% and 30%, respectively, after 24 h of treatment with 400 µg/mL EFW (Figure 1A).

However, even at the highest EFW concentration of 400 µg/mL, the growth of the lung carcinoma line A-549 and the bladder carcinoma line BFTC905 was not inhibited. When PBMCs were treated with 400 µg/mL EFW, over 50% of their growth survived (Figure 1B). These results showed that EFW selectively inhibited the growth of leukemic cancer cells, solid human cancer cells are not sensitive to EFW, and EFW has low toxicity to normal cells.

### 2.2. EFW Specifically Induces Cell Cycle Arrest

**Figure 2:**
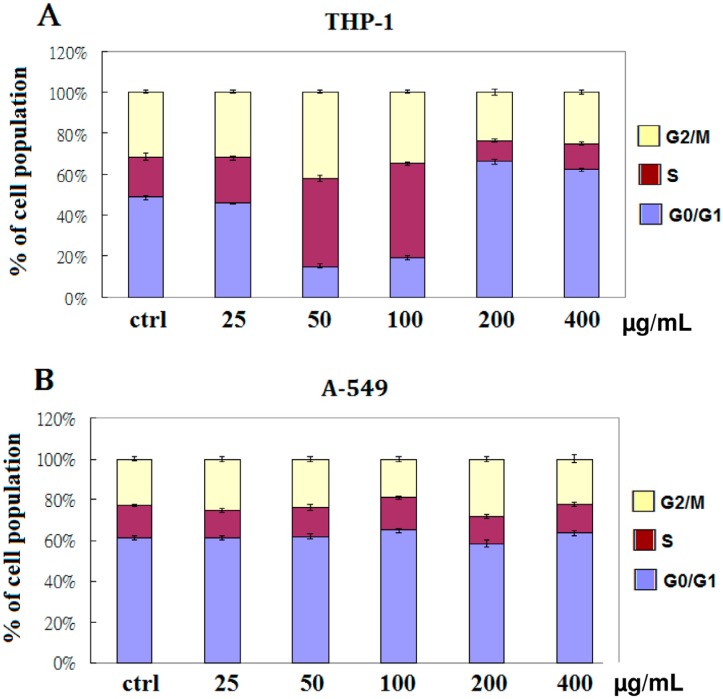
The effects of treatment with 25 μg/mL, 50 μg/mL, 100 μg/mL, 200 μg/mL, or 400 μg/mL of EFW or distilled water (control) for 24 h on the cell cycle were analyzed by examining the DNA content by FACS of (**A**) THP-1 and (**B**) A-549 cells to determine the percentage of cells in G2/M phase (yellow), S phase (red), and G0/G1 phase (blue). The results represent three independent experiments.

The cytotoxicity induced by EFW alters progression through the cell cycle of the leukemic THP-1 cells, as indicated by the significant increase in the percentage of cells in S phase from 17.7% without EFW to 42.8% at middle concentrations, and by the increase in cells in G0/G1 phase from 46.6% without EFW to 60.5% at higher concentrations (Figure 2). However, the lung carcinoma A-549 cells did not have significant changes in cell cycle distribution.

### 2.3. EFW Selectively Promoted Apoptosis for Leukemic Cells but not for Solid Human Cancer Cell Lines 

**Figure 3:**
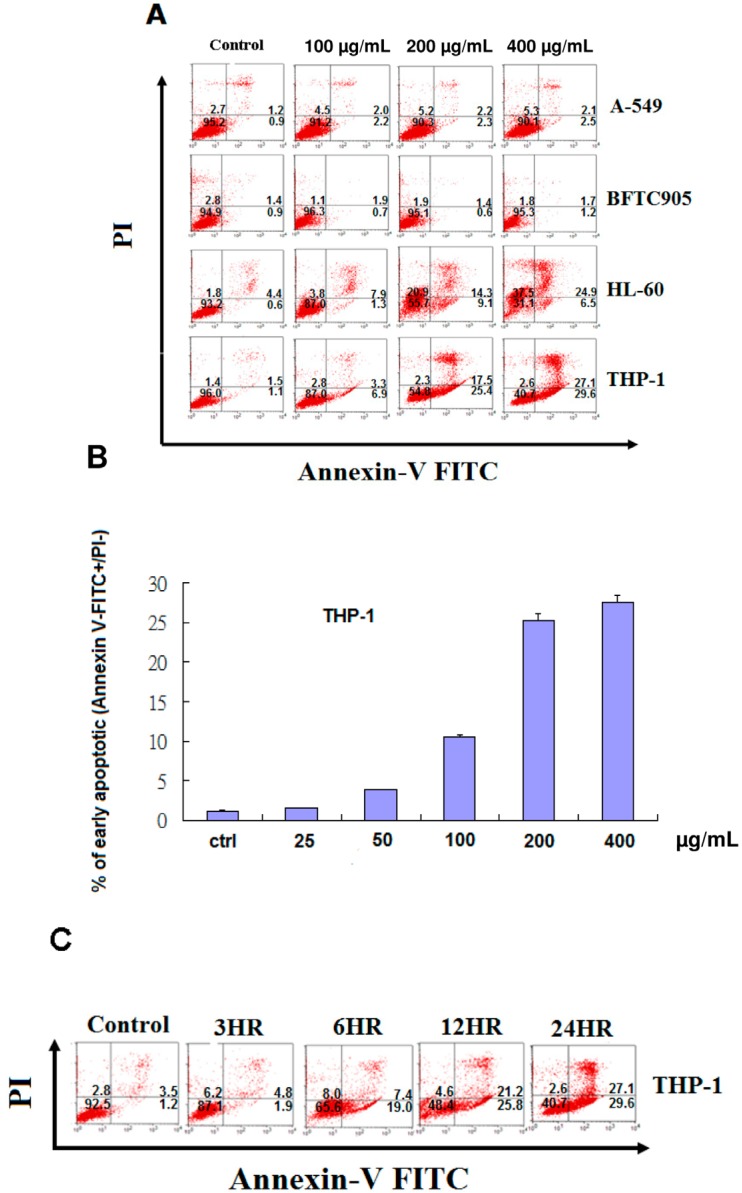
(**A**) The ability of EFW to induce apoptosis was examined by staining with Annexin V-FITC (x-axis) and PI (y-axis) of the A-549, BFTC905, HL-60, and THP-1 cell lines; (**B**) The effects of the dose of EFW were examined with doses of 25 μg/mL, 50 μg/mL, 100 μg/mL, 200 μg/mL, and 400 μg/mL EFW; (**C**) The effects of EFW over time on the THP-1 cells were examined by staining with Annexin V-FITC (x-axis) and PI (y-axis). The data represent the mean ± S.D. of three independent experiments.

We further verified that EFW induces apoptosis by examining the number of apoptotic cells by flow cytometry (Figure 3) after staining the different cell lines with FITC-Annexin V and PI. The numbers of both early and late apoptotic cells increased in a dose-dependent manner when THP-1 cells and HL-60 cells were treated with EFW at doses of 100 to 400 μg/mL (Figure 3A,B). When THP-1 cells were treated with EFW, the number of apoptotic cells significantly increased in a time-dependent manner (Figure 3c). The A-549 and BFTC905 cell lines did not have significant levels of apoptosis induced by EFW, even at 400 μg/mL (Figure 3A).

### 2.4. Apoptosis of THP-1 Cells by EFW via a Mitochondrial Pathway

**Figure 4:**
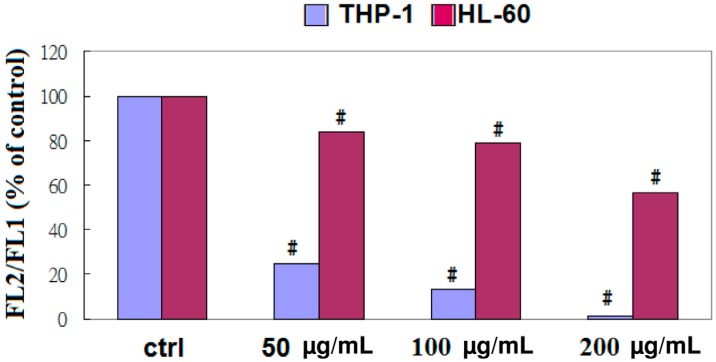
Mitochondrial membrane potential (ΔΨm) of THP-1 (blue) and HL-60 (red) cells was analyzed with cytofluorometry by double staining for JC-1 and PI after the cells were treated with 50 μg/mL, 100 μg/mL, or 200 μg/mL EFW for 24 h. The control was untreated cells. All of the experiments were performed at least three times, and all the results were obtained from three independent experiments. The symbol # indicates a significant difference between the treatment and control groups with *p* < 0.001.

The loss of mitochondrial membrane potential (ΔΨm) is an important event in apoptosis, so we evaluated ΔΨm values for the A-549, HL-60, and THP-1 cell lines after they were treated with EFW (Figure 4). The mitochondrial depolarization of the THP-1 and HL-60 cells after EFW treatments of 50 to 200 μg/mL indicated that EFW specifically regulated the apoptosis of leukemic cells via a mitochondrial pathway.

### 2.5. Apoptotic Pathways in THP-1 Cells Induced by EFW

**Figure 5:**
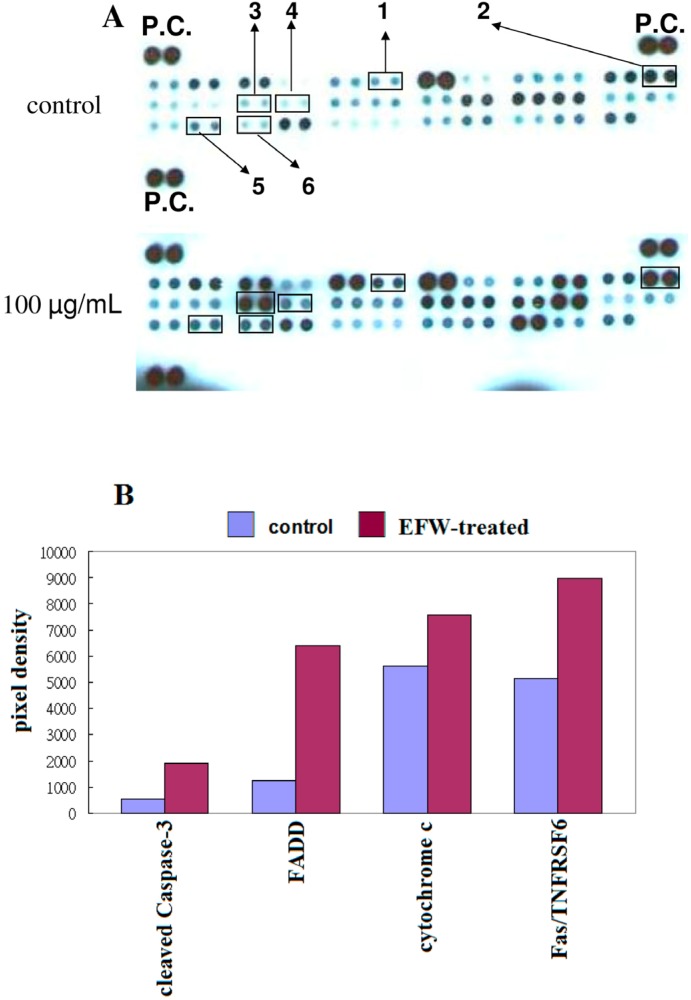
(**A**) The caspase-dependent apoptosis induced by treating THP-1 cells with 100 μg/mL EFW (red) or with no treatment (control; blue) for 24 h was examined by an apoptosis array analysis of whole cell lysates. The black underlines indicate (1) cleaved caspase-3, (2) cytochrome c, (3) FADD, (4) Fas/TNFRSF6, (5) p21, (6) p27, and (P.C.) positive control; (**B**) An image analyzer quantified the expression levels of the apoptosis-related proteins from THP-1 cells treated with 100 μg/mL EFW or with distilled water (control). Data represent mean ± S.D. of four measurements from two independent apoptosis array analyses.

We use a human apoptosis antibody array to evaluate the apoptotic pathways induced by EFW treatment by monitoring the expression of proteins related to apoptosis and to death receptor in THP-1 cells treated with EFW (Figure 5). We found significant increases in the expression of cleaved caspase-3 (from 2.4- to 3.7-fold), Fas-associated death domain-containing protein (FADD; from 2.2- to 5.1-fold), Fas/TNFRSF6 (from 1- to 1.7-fold), and cytochrome C (from 1.1- to 1.4-fold). In the THP-1 cells, the apoptosis induced by EFW used mechanisms that depend on caspase-3 in the THP-1 cells and that operate through the Fas/FADD and mitochondrial pathways.

## 3. Discussion

An aggressive malignancy with poor outcomes, AML is primarily a disease of the elderly, with a median age of onset of 72 years [13]. Remissions of AML come at the cost of considerable toxicity. In a recent report of older patients treated with standard therapy, 32% of the patients either died or could not receive additional therapy because of severe morbidity [14]. In elderly patients who do not die from treatment-related toxicity or resistant disease, remissions are short-lived, with median survival times of only about 12 months [13]. Despite decades of research, therapy has remained largely unchanged, with chemotherapy and bone marrow transplantation being the major treatments for AML. However, the high toxicity and serious complications caused by these conventional treatments has kept the survival rate for AML patients low. These problems have prompted us to search for novel agents with anticancer activity for AML treatment.

Apoptosis is a multi-step, multi-pathway program for cell death that is inherent in every cell of the body. Cancer alters the ratio of apoptosis to cell division. Successful treatment with chemotherapeutic drugs largely depends on their ability to trigger cell death in tumor cells, which at least partially involves activating apoptosis [15,16,17,18]. The apoptotic cascade can be initiated via two major pathways. The mitochondrial pathway involves releasing cytochrome c from the mitochondria, and the death receptor pathway involves activating death receptors in response to ligand binding [19,20,21,22]. When either pathway is triggered, the caspase family of cysteine proteases is activated, which executes the fate of the cell in a programmed fashion, leading to the typical morphologic changes [23]. The mitochondrial outer membrane is permeabilized in response to such intracellular stress signals as growth factor withdrawal, DNA damage, oxidative stress, or oncogene activation. The apoptotic signal is propagated by the consequent release of cytochrome c and other pro-apoptotic proteins. On the other hand, cell death by apoptosis is triggered when the membrane-bound Fas receptor (CD95, APO-1) is trimerized. The activation of Fas initiates a death pathway that involves a series of death-associated molecules [24], including FADD (Fas-associated death domain-containing protein). FADD is an adaptor protein that is recruited to the Fas receptor when the Fas receptor is engaged [25,26,27]. Then, FADD then binds to and activates procaspase-8 (also called FLICE or MACH) [26,27,28], which is believed to be the first step of a proteolytic cascade that triggers the activation of such other caspases as caspase-7, caspase-3, and caspase-6 [29]. Cytotoxic drugs used in cancer therapy can induce tumor cell death by apoptosis [16]. Some of them were shown to enhance the expression of Fas [30] and Fas-L [31]. Anticancer drug can induce Fas receptor clustering and FADD (Fas-associated death domain-containing protein) recruitment to Fas receptor in a Fas ligand-independent fashion [32].

Our present investigation indicated that both of the human leukemia cell lines treated with EFW underwent phytochemical-specific programmed cell death and apoptosis, and EFW did not induce non-specific necrotic death (Figure 3). Exposing human leukemia cell lines to EFW increased apoptosis in dose- and time-dependent manners. The pro-apoptotic effects of EFW on human leukemia cells are exerted by inducing a loss of the mitochondrial membrane potential, the release of cytochrome C, and increasing levels of FADD and Fas/TNFRSF6 proteins. Apoptosis is induced by EFW in leukemic cancer cells through different cell signaling pathways including the Fas and mitochondrial pathways. These data strongly support further investigations of EFW as a novel candidate for an anticancer drug. Future studies will evaluate the antitumor activity of EFW in animal models. 

Medicinal plants are important sources for developing effective anticancer agents. In fact, more than half of the today’s anticancer drugs were originally from natural products and their derivatives. Recently, it has already been reported that medicinal plant-derived molecules may induce apoptosis in leukemic cancer cells [33,34]. Clinically and pathologically, leukemia is subdivided into a variety of groups. Most forms of leukemia are treated with pharmaceutical medication, such as cytarabine is mainly used in the treatment of acute myeloid leukemia, acute lymphocytic leukemia (ALL) and in lymphomas, Etoposide is used as a form of chemotherapy for lymphoma, nonlymphocytic leukemia. However, none of the drugs is active against all forms of leukemia. Therefore, searching for new reagents to treat leukemia is necessary. We tested 20 extracts derived from Taiwanese medical plants and 48 synthetic chemicals in leukemic cells. The most striking apoptosis-inducing effect was observed in an extract prepared from *Euphorbia formosana* Hayata (EF), and it was the only one which could potently and specifically induced apoptosis in leukemic cell lines.

Our *in vitro* cytotoxic assays found that an extract from *Euphorbia* *formosana*, a Taiwanese native medicinal plant, selectively inhibits human leukemic cancer cell growth. Furthermore, the same experiments showed that EFW treatment was less toxic to normal PBMCs. Thus, we sought to elucidate the function and molecular mechanisms of EFW in inhibiting tumor cell growth so that we can understand and better use this traditional Chinese medicine in cancer therapy.

## 4. Experimental 

### 4.1. Preparing Water Extracts from Euphorbia formosana

The dried roots of EF were purchased from Hualien, Taiwan in August 2008 and identified by one of the authors (Y.-L.L.). The root was boiled in water and brought back to room temperature a total of three times. The liquids were collected and lyophilized. A stock solution of the EF extract in water (EFW) was prepared by suspending lyophilized material in water and passing it through a 0.22 μm filter. Working solutions were prepared by mixing the stock solution with a cell culture medium.

### 4.2. Cell Lines and Culture

We purchased the human lung carcinoma cell line A-549, the human bladder papillary transitional cell carcinoma cell line BFTC905, the human monocytic leukemia-derived cell line THP-1, and the human promyelocytic leukemia cell line HL-60 from Food Industry Research and Development Institute (Hsinchu, Taiwan). Peripheral blood mononuclear cells (PBMCs) were obtained from healthy blood donors. Cells were grown in media according to the instructions of the suppliers, supplemented with 10% fetal bovine serum (Gibco Invitrogen, Carlsbad, CA, USA), 100 IU/mL penicillin, and 100 IU/mL streptomycin in a humidified atmosphere with 5% CO_2_ at 37 °C.

### 4.3. Reagents

All reagents were purchased from Sigma-Aldrich (St. Louis, MO, USA) unless otherwise stated. The human apoptosis antibody array kit (ARY009) was purchased from R&D Systems (Minneapolis, MN, USA). The CD14 MicroBeads kit was purchased from Miltenyi Biotec GmBH (Bergisch Gladbach, Germany). The annexin V-FITC apoptosis detection kit was purchased from Calbiochem (Merck KGaA, Darmstadt, Germany).

### 4.4. Cell Viability Assay

The percentage of growth inhibition was analyzed by flow cytometry to measure viable cells. A total of 3 × 10^6^ cells were seeded onto 6 cm culture dishes for 16 h. Next, the cell lines and PBMCs were treated with 25 μg/mL, 50 μg/mL, 100 μg/mL, 200 μg/mL, or 400 μg/mL concentrations of EFW. The cells were incubated for between 3 and 24 h at 37 °C, washed twice with PBS after incubating for 3 to 24 h at 37 °C, then, resuspended in 500 μL staining solution containing 1.25 μL FITC-conjugated annexin V (Calbiochem) and 10 μL propidium iodide (PI). After 5 min at room temperature, approximately 10,000 cells from each sample were analyzed by a flow cytometry. Each experiment was performed in triplicate. The distributions of viable cells (annexin V-FITC-/PI) were determined by the Cell Quest software (Becton Dickinson, Oxford, UK).

### 4.5. Cell Cycle Analysis

Cells were cultured in 6 cm culture dishes, treated with the various concentrations of EFW for 24 h, and then washed twice with cold PBS solution. The floating and adherent cells were collected, fixed, and permeabilized with cold 70% ethanol at 4 °C overnight. The cell mixture was then incubated in 1 mL of cold PI stain solution containing 20 mg/mL PI, 10 mg/mL RNase A, and 5% Triton X-100 for 30 min in the dark at room temperature. Next, the cell cycle was analyzed by flow cytometry, with the cell cycle distribution analyzed with Cell Quest software. Each experiment was conducted three times. 

### 4.6. Human Apoptosis Antibody Array

Apoptosis-related proteins were detected and analyzed with a human apoptosis array kit (R&D Systems), which contains duplicate spots of 35 apoptosis-related proteins. Briefly, the membrane containing immobilized apoptosis-related antibodies was blocked with bovine serum albumin for 1 h on a rocking platform at room temperature. The membrane was then incubated overnight at 2 °C to 8 °C on a rocking platform with lysates of THP-1 cells, with or without EFW, and with detection antibody cocktail. The membrane was incubated with streptavidin-horseradish peroxidase conjugate followed by chemiluminescent detection reagent. The membrane was scanned and its pixel density was calculated by quantifying the mean spot densities from two independent experiments. 

### 4.7. Isolating PBMCs and Purification of Lymphocytes and Monocytes

Peripheral blood mononuclear cells (PBMCs) were obtained from healthy blood volunteers and were prepared by density gradient centrifugation of blood over Histopaque-1077 (Sigma-Aldrich). PBMCs were separated into CD14+ monocytes (M-PBMC) and lymphocyte (L-PBMC) subsets by immunomagnetic selection with the CD14 MicroBeads kit (Miltenyi Biotec GmBH, Bergisch Gladbach, Germany). After immune-magnetic selection with anti-CD14 antibodies, the purity of the monocytes prepared from PBMCs was over 95%, as analyzed by flow cytometry. A highly enriched population of lymphocytes was obtained from the CD14-depleted cell fraction. Cell preparations were aliquoted to 1 × 10^6^ cells/mL in RPMI 1640 medium (Gibco Invitrogen) supplemented with 10% fetal calf serum (Gibco Invitrogen), 0.1 mg/mL of streptomycin sulfate (Sigma-Aldrich), 0.1 mg/mL of penicillin (Gibco Invitrogen) and 100 mM L-glutamine (Sigma-Aldrich). Cells were cultured at 37 °C in a humidified atmosphere with 5% CO_2_.

### 4.8. Detecting Apoptotic Cells with Annexin V-FITC and Propidium Iodide Staining

Cells were stained with annexin V-FITC and/or PI based on the protocol of the annexin V-FITC apoptosis detection kit (Calbiochem). Lymphocytes, monocytes, A-549, BFTC905, HL-60, and THP-1 cells (10^6^ per test) were incubated with EFW concentrations of 25 to 400 µg/mL, washed twice with PBS, and then resuspended in 500 μL staining solution containing 1.25 μL FITC-conjugated annexin V (Calbiochem) and 10 μL PI. The cells were incubated at room temperature for 5 min and then about 10,000 cells from each sample were analyzed by flow cytometry. The flow cytometry data was analyzed by FACScan (Becton-Dickinson). Cells that were apoptotic, necrotic, or both were identified by staining with annexin V-FITC and PI. The percentages of distribution of normal (annexin V-FITC−/PI−), early apoptotic (annexin V-FITC+/PI−), late apoptotic or necrotic (annexin V-FITC+/PI+) and necrotic cells (annexin V-FITC−/PI+) were calculated by the Cell Quest software.

### 4.9. Assessment of Mitochondrial Membrane Potential (ΔΨm)

The change in mitochondrial membrane potential (ΔΨm) was measured using the lipophilic cation JC-1 (5,5',6,6'-tetrachloro-1,1',3,3'-tetraethylbenzimidazolyl carbocyanine iodide, stock: 5 mg/mL). The accumulation of JC-1 in mitochondria is potential-dependent and is indicated by a fluorescence emission shift from green (JC-1 in monomeric form, 527 nm) to red (JC-1 in aggregative form, 590 nm). Consequently, mitochondrial depolarization is indicated by a decrease in the intensity ratio of red to green fluorescence. The A-549, HL-60, and THP-1 cells at a concentration of 5 × 10^5^ cells/mL were incubated in 6-well plates for 24 h at 37 °C with 5% CO_2_ either with EFW at concentrations from 25 to 400 μg/mL or without EFW. Next, the cells were washed twice with PBS, stained with 1 mM JC-1 in dimethyl sulfoxide (DMSO, Sigma) for 30 min in the dark at 37 °C, and then immediately analyzed with a Becton-Dickinson FACSan flow cytometer at 488 nm excitation. The data was analyzed with CellQuest Pro software.

### 4.10. Statistical Analysis

All experiments were performed at least in triplicate. Data are presented as mean ± standard deviation (SD), and *p*-values were analyzed with the Student’s *t*-test, accompanied by analysis of variance (ANOVA) where appropriate.

## 5. Conclusions

The pronounced *in vitro* anticancer activity of EFW suggests potent anticancer effects that cause both cell cycle arrest and apoptosis of leukemic cancer cells. These results concerning antileukemic treatment with EF are significant, since they provide new hope for effective therapeutics to manage leukemic cancer cells. A total of 35 compounds from *Euphorbia formosana* have been isolated and purified by high-pressure liquid chromatography and spectroscopy to elucidate their structures in our laboratory. These compounds include 14 polyphenols, four steroids, one peptide, one furan, three coumarins, four diterpenes, four triterpenes, two flavonoids, and others (Table 1). We are further analyzing the specific active compounds of EFW, examining their mechanisms of action, and conducting *in vivo* studies. This work may lead to new therapeutic options and improved understanding of how phytochemicals interact to induce apoptosis in leukemia cancer cells.

## Figures and Tables

**Table 1:** Compounds purified from *Euphorbia formosana*.

**I. Polyphenols**		
Ellagic acid	3,3'-Di-*O*-methylellagic acid	3,3',4,4'-Tetra-*O*-methylellagic acid
3,3'-Di-*O*-methylellagic acid-4'-*O*-b-xylopyranoside	3,3'-Di-*O*-methylellagic acid-4'-*O*-b-glucoside	3,3'-Di-*O*-methylellagic acid-4'-*O*-b-arabinopyranoside
3'-*O*-methyl-3,4-methylenedioxyellagic acid	Gallic acid	Methyl gallate
Methyl brevifolincarboxylate	Brevifolin	Phyllanthusiin E
Dehydrochebulic acid trimethyl ester	Octacosyl ferulate	
**II. Steroids**		
β-Sitosterol	β-Sitosteryl-3-*O*-glucoside	β-Sitostenone
Ergosterol peroxide		
**III. Peptide**		
Aurantiamide acetate		
**IV. Furan**		
5-Hydroxymethylfurfural		
**V. Coumarins**		
Scopoletin	Euoniside	6-Methoxy-7,8-methylenedioxycoumarin
**VI. Diterpenes**		
Helioscopinolide E	Isopimara-7,15-dien-3-one	Epi-manool
Larixol		
**VII. Triterpenes**		
Euphol	Glutinone	Cycloart-23-ene-3b,25-diol
Tirucalla-8,25-diene-3,24-diol		
**VIII. Flavonoids**		
Quercetin-3-*O*-α-L-rhamnoside	Kaempferol-3-*O*-α-l-rhamnoside	
**IX. Others**		
4-Methyl-5,6-dihydropyran-2-one	1,3,4,6-tetra-*O*-galloyl-β-d-glucopyranose	
